# The Association between Serum 25-Hydroxyvitamin D3 Levels and Pro-Inflammatory Markers in New-Onset Type 2 Diabetes Mellitus and Prediabetes

**DOI:** 10.3390/biom13121778

**Published:** 2023-12-12

**Authors:** Aysen Kutan Fenercioglu, Mustafa Sait Gonen, Hafize Uzun, Nurver Turfaner Sipahioglu, Gunay Can, Ebru Tas, Zehra Kara, Hande Mefkure Ozkaya, Pinar Atukeren

**Affiliations:** 1 Department of Family Medicine, Cerrahpasa Faculty of Medicine, Istanbul University-Cerrahpasa, 34320 Istanbul, Turkey; nurver@iuc.edu.tr (N.T.S.); ebrubicer92@gmail.com (E.T.); 2 Department of Internal Medicine, Division of Endocrinology and Metabolism, Cerrahpasa Faculty of Medicine, Istanbul University-Cerrahpasa, 34320 Istanbul, Turkey; m.saitgonen@hotmail.com (M.S.G.); drzehrakara@yahoo.com (Z.K.); hndebektas@gmail.com (H.M.O.); 3 Department of Medical Biochemistry, Faculty of Medicine, Istanbul Atlas University, 34403 Istanbul, Turkey; huzun59@hotmail.com; 4 Department of Public Health, Cerrahpasa Faculty of Medicine, Istanbul University-Cerrahpasa, 34320 Istanbul, Turkey; gunaycan09@yahoo.fr; 5 Homecare Unit, Isparta Sehit Yunus Emre State Hospital, 32300 Isparta, Turkey; 6 Department of Medical Biochemistry, Cerrahpasa Faculty of Medicine, Istanbul University-Cerrahpasa, 34320 Istanbul, Turkey; p_atukeren@yahoo.com

**Keywords:** prediabetes, type 2 diabetes mellitus, 25-hydroxyvitamin D3, inflammation, interleukins, TNF-α, NF-kB, MAPK

## Abstract

In this study, we aimed to reveal the pro-inflammatory effects of serum 25-hydroxyvitamin D3 (Vit D) deficiency and insufficiency in new-onset type 2 diabetes mellitus (T2DM) and prediabetes. We recruited 84 prediabetes patients, 94 new-onset T2DM patients and 113 healthy participants. We measured the levels of C-reactive protein (CRP), fibrinogen, ferritin, interleukin-1β (IL-1β), interleukin-6 (IL-6), interleukin-8 (IL-8), tumor necrosis factor-α (TNF-α), nuclear factor kappa-B (NF-κB) and mitogen-activated protein kinase (MAPK) in the serum of the participants. ANOVA Bonferroni and Kruskal–Wallis Dunn tests were used to compare the inflammation markers and vitamin D levels between the groups. Based on covariance analysis with age, gender and BMI, the Vit D levels of the T2DM group were significantly lower (*p* < 0.003). Pro-inflammatory markers and CRP were significantly higher in prediabetic and diabetic subjects. In the prediabetes group, IL-1β, IL-6, IL-8, TNF-α and MAPK were significantly higher in those with Vit D insufficiency and deficiency groups. In the T2DM group, IL-1β, IL-6, IL-8, TNF-α, NF-κB, MAPK and CRP were significantly higher in those with Vit D insufficiency and deficiency. Our study emphasizes the pro-inflammatory effects of Vit D deficiency and insufficiency in new-onset type 2 diabetes mellitus and prediabetes.

## 1. Introduction

Inflammation is defined as a host-induced reaction, especially against infections and tissue damage; however, recent studies revealed that it also plays a role in the pathophysiology of chronic diseases. In the last fifteen years, type 2 diabetes mellitus (T2DM) has been described as a chronic subclinical inflammation state associated with an acute phase response [1]. Scientific studies have shown that increased pro-inflammatory biomarkers, such as tumor necrosis factor-α (TNF-α), interferon-gamma, interleukin-1 beta (IL-1ß), interleukin-6 (IL-6), high-sensitivity C-reactive protein (hs-CRP) and mitogen-activated protein kinases (MAPK), were associated with T2DM [1,2].

Today, vitamin D (Vit D) deficiency is a global issue. Although its relationship with osteoporosis is very well-known, Vit D deficiency is associated with many other diseases such as autoimmune diseases, various types of cancer, cardiovascular diseases and T2DM. It has been linked to the development of hyperglycemia by altering insulin secretion [3,4]. Hypovitaminosis D elevates intracellular calcium levels by increasing the secretion of the parathyroid hormone, which in turn inhibits calcium-related insulin secretion and action [5]. In addition, the role of serum Vit D in the development of diabetes-related cardiovascular and renal complications has been demonstrated in many studies [6,7,8].

Furthermore, in vitro and in vivo studies revealed that 1,25-dihydroxyvitamin D3 (1,25(OH)2D3), the biologically active form of Vit D, has various immunomodulatory functions. Some of these functions are the suppression of pro-inflammatory cytokines, the regulation of cellular immunity, the suppression of B-cell differentiation and maturation to plasma cells, the decrease in the expression of nuclear factor kappa-B (NF-κB) and the downregulation of MAPK [9,10,11]. Through all these positive effects on the immune system and inflammation, Vit D reduces insulin resistance and increases insulin secretion in patients with T2DM [1,2].

NF-κB is a transcription factor found in the cytoplasm of all cell types. When activated, it is transported to the nucleus and regulates the gene expression of cytokines and adhesion molecules; furthermore, it controls cell apoptosis, adaptive immunity, cell growth and aging [12,13,14,15]. Because it is activated by pro-inflammatory cytokines such as IL-1β and TNF-α and is involved in the synthesis of genes of cytokines, chemokines and adhesion molecules, NF-κB has been recognized as a marker of inflammation for a while [15]. The number of scientific studies emphasizing that Vit D suppresses the release of IL-6, IL-8 and TNF-α from mononuclear cells through NF-κB or MAPK has increased over time [9,11].

The present study aimed to show the pro-inflammatory effects of Vit D deficiency and insufficiency on inflammation in new-onset type 2 diabetes mellitus (T2DM) and prediabetes. For this purpose, we assessed the relationship between serum Vit D levels and acute phase reactants such as CRP, fibrinogen and ferritin and pro-inflammatory markers such as IL-1β, IL-6, IL-8, TNF-α, NF-κB and MAPK in newly diagnosed prediabetes and type 2 DM patients.

## 2. Materials and Methods

### 2.1. Ethical Approval

All subjects who participated in this study signed written informed consent before participating. The Ethics Committee of Istanbul University-Cerrahpaşa, Cerrahpaşa Medical Faculty, (approval date and number 16.04.2020-54969) approved this study. This study was conducted according to the Declaration of Helsinki.

### 2.2. Research Design

This is a cross-sectional study conducted in the outpatient clinics of the Endocrinology and Family Medicine Departments of Cerrahpaşa Medical Faculty, Istanbul University-Cerrahpaşa, between May 2020 and May 2022. A total of 291 participants aged 30–60 years were recruited for this study. The study groups consisted of 84 prediabetes (PreDM) patients and 94 T2DM patients who were newly diagnosed and had no diabetes complications. The control group consisted of 113 healthy participants without any chronic disease. According to the American Diabetes Association Prediabetes Diabetes Mellitus classification, participants whose fasting blood glucose (FBG) was between 100 mg/dL (5.6 mmol/L) and 126 mg/dL (7.0 mmol/L) were recruited in the prediabetes group; participants whose FBG level was >126 mg/dL (7.0 mmol/L) were recruited in the diabetes group; and participants with FBG level < 100 mg/dL (5.6 mmol/L) were considered healthy.

The preDM and diabetes (DM) groups were further divided into the normal level Vit D group, Vit D insufficiency group and Vit D deficiency group. In line with the recommendations of the US Society of Endocrinology, Vit D levels below 20 nmol/L were considered deficient; levels between 20.01 nmol/L and 29.99 nmol/L were considered insufficient; and 30 nmol/L and above were considered normal vitamin D levels.

### 2.3. Study Participants

The subjects consisted of patients and healthy individuals who met the inclusion criteria and agreed to participate in the study. The study group consisted of DM and preDM patients recruited from outpatient clinics in the Endocrinology and Family Medicine Departments. The control group (nonDM group) consisted of healthy individuals who applied to the outpatient clinics of the Family Medicine Department for general health examinations. The nature of the research was explained to the participants, and written informed consent was obtained from them after they agreed to participate in this study.

### 2.4. Inclusion Criteria

Individuals between 30 and 60 years of age who were mentally competent, not pregnant and did not have diabetes complications, infections, connective tissue disorders or malignancies were included in this study. As aging increases inflammation, individuals over 60 years of age were not included in this study.

### 2.5. Exclusion Criteria

Considering their effects on the pro- and anti-inflammatory parameters, healthy participants with a history of any chronic disease, healthy and diabetic participants who used steroids, anti-inflammatory drugs, antiepileptic drugs or antioxidant drugs and those who used vitamin D preparations, calcium preparations or other vitamin preparations in the past three months, smokers and alcohol-dependent individuals were excluded from this study.

### 2.6. Study Design

Blood samples were obtained from patients by venipuncture after an overnight fast (≥8 h). Serum samples were obtained after at least 30 min of clotting by centrifugation at 2500× *g* for 15 min and stored at −80 °C until they were assayed for the determination of IL-1β, IL-6, IL-8, TNF-α, NF-κB and MAPK. Routine biochemical parameters and Vit D3 were measured on the day of blood collection.

The biochemical parameters were measured using the spectrophotometric method with an autoanalyzer (Hitachi Modular System, Roche Diagnostic, Corporation, Hague Road, Indianapolis, IN, USA). The CRP values were measured using the turbidimetric method with an autoanalyzer (ADVIA 1800 Auto Analyzer, Siemens Medical Sol., Deerfield, IL, USA). Using a Fibrintimer II coagulometer and Multifibren U Kit (Siemens Healthcare Diagnostics, Marburg, Germany), the Clauss method was performed to measure plasma fibrinogen. The 25-hydroxyvitamin D3 (25(OH)D3) levels were assessed using an immunoassay based on electrochemiluminescence (ECL) technology.

Serum IL-1β levels were measured according to the manufacturer’s instructions (Human IL-1β ELISA Kit, Cat.No.:E-EL-H0149, Elabscience, Houston, TX, USA). The coefficients of intra- and interassay variation were <8% (*n* = 20) and <9% (*n* = 20), respectively.

Serum IL-6 levels were measured according to the manufacturer’s instructions (Human IL-6 ELISA Kit, Cat.No.:E-EL-H6156, Elabscience, Houston, TX, USA). The coefficients of intra- and interassay variation were <8% (*n* = 20) and <9% (*n* = 20), respectively.

Serum IL-8 levels were measured according to the manufacturer’s instructions (Human IL-8 ELISA Kit, Cat.No.:E-EL-H6008, Elabscience, Houston, TX, USA). The coefficients of intra- and interassay variation were <8% (*n* = 20) and <9% (*n* = 20), respectively.

Serum TNF-α levels were measured according to the manufacturer’s instructions (Human Tumor Necrosis Factor Alpha (TNF-α) ELISA Kit, Cat.No.:E-EL-H0109, Elabscience, Houston, TX, USA). The coefficients of intra- and interassay variation were <8% (*n* = 20) and <9% (*n* = 20), respectively.

Serum NF-κB-p65 levels were measured according to the manufacturer’s instructions (Human NFKB-p65 (Nuclear factor NF-kappa-B p65) subunit ELISA Kit, Cat.No.:E-EL-H1388, Elabscience, CA, USA). The coefficients of intra- and interassay variation were <8% (*n* = 20) and <9% (*n* = 20), respectively.

Serum p38 mitogen-activated protein kinase (MAPK) levels were measured according to the manufacturer’s instructions (Human MAPK ELISA Kit, Cat.No.:CSB-E09147h, CUBIO, Houston, TX, USA). The coefficients of intra- and interassay variation were <8% (*n* = 20) and <9% (*n* = 20), respectively.

### 2.7. Statistical Analysis

The Epi Info™ StatCalc. program was used for the power analysis in our study. While determining the number of subjects, we used the frequency of Vit D deficiency/insufficiency. With an expected frequency of 20% of Vit D deficiency/insufficiency in the nondiabetes group and 40% of Vit D deficiency/insufficiency in the diabetes group, with a type 1 error of 0.05, a 95% confidence interval and 80% power, a sample size of 81 subjects in both groups was calculated. To address the possibility of participants leaving this study without giving blood, the sample size was increased by 15%. The data were analyzed using SPSS for Windows version 16.0 (SPSS Inc.; Chicago, IL, USA).

Continuous variables are presented as means ± standard deviation (SD). The differences in continuous and categorical variables between the groups were separately evaluated using ANOVA Bonferroni and Kruskal–Wallis Dunn tests. Covariance analysis for gender, BMI and age were performed by pairwise comparisons. The Pearson and Spearman correlation tests was used to evaluate the relationship between the parameters in the analyses. Logistic regression analysis was used to determine the relationships between anti-inflammatory markers and Vit D levels in the DM group.

## 3. Results

In our study, Vit D levels were higher in the nonDM group compared to the DM group (*p* = 0.001) and in the preDM group compared to the DM group (*p* = 0.014) (Table 1). While there was no significant difference in the serum calcium levels, the parathormone (PTH) level was higher in the DM group compared to the preDM group due to the difference in Vit D levels (*p* = 0.034). Covariance analysis with age, gender and BMI revealed that the Vit D levels in the DM group were significantly lower than those in the preDM and nonDM groups (*p* < 0.003). Serum IL-1β, IL-6, IL-8, TNF-α, NF-κB and MAPK levels and acute phase reactants such as CRP, fibrinogen and ferritin were statistically significantly higher in the DM group than in the nonDM group. Serum IL-1β, IL-6, TNF-α, NF-κB and MAPK levels and CRP were found to be statistically significantly higher in the preDM group than in the nonDM group (Table 1). When the preDM group was compared with the DM group, TNF-α, NF-κB and MAPK levels and CRP, fibrinogen and ferritin were found to be statistically significantly higher in the DM group than in the preDM group (Table 1).

The subjects’ FBG levels were found to be lower in those with normal Vit D levels than in those with deficient Vit D levels (*p* = 0.00). Serum IL-1β, IL-6, IL-8, TNF-α and MAPK levels and CRP were found to be statistically significantly lower in patients with normal Vit D levels compared to those with insufficient Vit D levels. Serum IL-1β, IL-6, IL-8, TNF-α, NF-κB, MAPK and CRP levels were found to be statistically significantly lower in patients with normal Vit D levels compared to those with deficient Vit D levels. When the Vit D-deficient group was compared with the Vit D-insufficient group, IL-1β, IL-6, IL-8, TNF-α and NF-κB were found to be statistically significantly higher in the Vit D-deficient group (Table 2).

**Figure 1:**
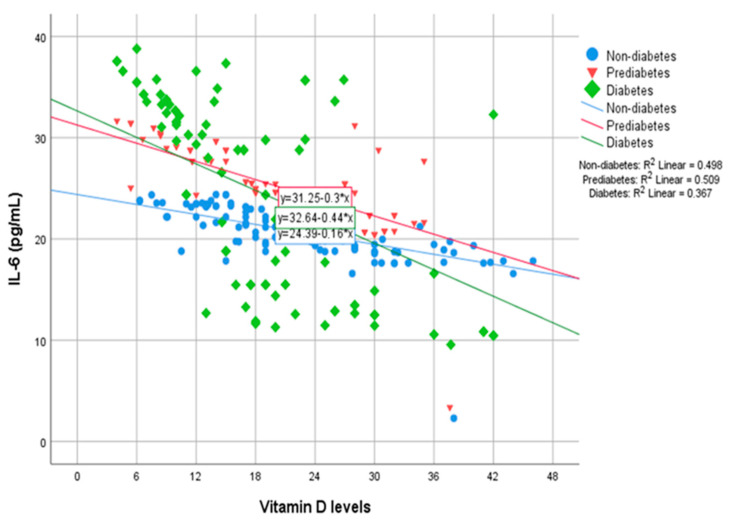
Distribution of IL-6 values by Vit D levels in all groups.

In the preDM group, IL-1β, IL-6, IL-8, TNF-α and MAPK levels were found to be statistically significantly higher in those with Vit D insufficiency and deficiency compared to those with normal Vit D levels (Table 3). In this group, covariance analysis with age, gender and BMI revealed that IL-1β was statistically significantly higher in Vit D insufficiency and deficiency groups compared to the normal Vit D group (*p* = 0.048 and *p* = 0.00); IL-6 and IL-8 were statistically significantly higher in the Vit D deficiency group compared to the normal Vit D group (*p* = 0.00 and *p* = 0.00) (Figure 1); TNF-α was statistically significantly higher in the Vit D insufficiency and deficiency groups compared to the normal Vit D group (*p* = 0.019 and *p* = 0.00); and MAPK was statistically significantly higher in the Vit D deficiency group compared to the normal Vit D group (*p* = 0.00). Covariance analysis of acute phase reactants with age, gender and BMI did not reveal any statistical significance in the preDM group.

In the DM group, IL-1β, IL-6, IL-8, TNF-α, NF-κB, MAPK and CRP levels were statistically significantly higher in those with Vit D insufficiency and deficiency compared to those with normal Vit D levels (Table 4). In this group, covariance analysis with age, gender and BMI revealed that IL-1β and IL-8 were statistically significantly higher in the Vit D deficiency group compared to the normal Vit D group (*p* = 0.00 and *p* = 0.004); IL-6 was statistically significantly higher in the Vit D deficiency and insufficiency groups compared to the normal Vit D group (*p* = 0.00 and *p* = 0.044) (Figure 1); TNF-α was statistically significantly higher in the Vit D insufficiency and deficiency groups compared to the normal Vit D group (*p* = 0.002 and *p* = 0.00); NF-κB was statistically significantly higher in the Vit D deficiency group compared to the normal Vit D group (*p* = 0.009); and MAPK was statistically significantly higher in the Vit D deficiency group compared to the normal Vit D group (*p* = 0.005). Covariance analysis of acute phase reactants with age, gender and BMI did not reveal any statistical significance in the DM group.

The correlation analyses between groups revealed a statistically significant negative correlation between IL-1β, IL-6, IL-8, NF-κB and MAPK levels and Vit D levels in all groups (Table 5). In addition, a significant negative correlation between CRP and Vit D levels existed in the diabetes group (r = 0.258) (Table 5).

The logistic regression analysis revealed a statistically significant relationship between diabetes and age, low Vit D levels, high TNF-α and high MAPK levels (Table 6).

## 4. Discussion

This study revealed that the IL1-β, IL-6, IL-8, TNF-α, NF-κB and MAPK levels as pro-inflammatory markers and CRP, fibrinogen and ferritin as serum acute phase reactants were significantly increased in the prediabetes and T2DM groups compared to nondiabetes group (Table 1). In addition, the TNF-α, NF-κB and MAPK levels and acute phase reactants were significantly higher in the diabetes group compared to the prediabetes group. In both groups, covariance analysis with age, gender and BMI revealed that those with deficient and insufficient serum Vit D levels had higher levels of cytokines and inflammatory markers than those with normal serum Vit D levels. The significance of this study is the effects of serum Vit D levels on the development of inflammation in both prediabetes and diabetes. In particular, the effects of serum Vit D levels on MAPK and NF-κB are of great importance because these two markers stimulate the synthesis of other pro-inflammatory markers.

There are Vit D receptors on the β-cells of the pancreas, and animal studies have shown that insulin secretion is increased after the addition of 1,25(OH)2D3 to the medium [16,17]. Increasing intracellular calcium also increases insulin secretion from these cells. Studies have demonstrated an increased calcium-binding protein in these cells as evidence of this mechanism. On the other hand, Vit D deficiency causes secondary hyperparathyroidism, which in turn inhibits calcium-related insulin secretion [5]. In patients with T2DM and the general population, lower Vit D levels are associated with higher FBG, insulin resistance and metabolic syndrome as revealed in the Amsterdam Longitudinal Aging study [18]. Since Vit D deficiency is associated with increased pro-inflammatory markers, another mechanism leading to insulin resistance and diabetes is increased inflammation [1,2,17]. In addition, genetic polymorphisms of the Vit D receptor or Vit D binding protein (DBP) may predispose someone to T2DM. DBP polymorphisms were associated with higher fasting plasma insulin in a study conducted in Japan [16].

In the Women’s Health Initiative Calcium and Vitamin D study, 33,951 postmenopausal women were randomized to receive 400 IU/day vitamin D3 and 1000 mg/day calcium or a double placebo for 7 years, and it was found that the risk of developing T2DM was reduced [19]. The improvement in insulin resistance occurs mostly when the serum 25-hydroxyvitamin D3 (25(OH)D3) level is 80 nmol/L and above [20]. In the Third National Health and Nutrition Evaluation Study (NHANES III), conducted on 6228 individuals, 25(OH)D3 was found to protect against the development of T2DM above 81 nmol/L [21]. On the other hand, in a multicenter randomized placebo-control study involving 2423 participants who were at high risk for T2DM, Vit D supplementation of 4000 IU/day did not result in a significantly lower risk of diabetes than the placebo after a follow-up period of 2.5 years [22]. However, the participants in the study were not selected for Vit D insufficiency, and participants with BMI > 30 were also included in the study. In the study conducted by Al-Ghadeer et al. in Saudi Arabia, hemoglobin A1c (HbA1c) was above 5.9 in 71.9% of those with Vit D deficiency; this rate was 25% for those with Vit D insufficiency and only 2.2% for those with normal Vit D levels [23]. In our study, the FBG, HbA1c and insulin levels were higher in the Vit D deficiency group compared to the normal Vit D group (Table 2). In addition, serum Vit D levels were found to be lower in the DM group compared to the nonDM group and preDM group (Table 1).

High glucose concentrations activate reactive oxygen species (ROS) and NF-κB pathways, inducing the secretion of the inflammatory cytokine monocyte chemotactic protein-1 (MCP-1) and leukocyte interleukin-8 (IL-8). High serum MCP-1 and IL-8 levels accelerate the progression of diabetes. The randomized controlled study by Gu et al. in China showed that the serum MCP-1 and IL-8 levels decreased in diabetics who took 400 IU of Vit D daily [24]. NF-κB is also involved in the synthesis of genes of cytokines and promotes B lymphocyte maturation [12,13,14,15]. In our study, the correlation analyses between groups revealed a statistically significant negative correlation between IL-1β, IL-6, IL-8, NF-κB and MAPK levels and Vit D levels in both DM and preDM groups (Table 5).

T2DM patients usually have significantly elevated serum ferritin levels or iron status, and people with high ferritin levels have a higher risk of developing T2DM. Excess iron intake, especially heme iron from red meat, increases the risk of developing T2DM through oxidative damage to beta cells and impaired hepatic glucose production suppression. Pancreatic β-cell iron uptake was believed to be facilitated by divalent metal transporter 1 (DMT1), and the DMT1 expression was regulated by NF-κB activity. The study by Zhao et al. in diabetic rats showed that Vit D could improve islet morphology and β-cell function by attenuating iron accumulation. These effects were attributed to the suppression of the NF-κB-DMT1 pathway as a result of vitamin D supplementation [25]. In our study, ferritin and NF-κB levels were found to be lower in the DM group with normal Vit D levels compared to those with deficient Vit D levels, but no statistically significant correlation was found for ferritin levels. While the plasma ferritin levels reflect iron stores, they are also determined by many other genetic and environmental factors. Therefore, it is possible that the relationship between ferritin and diabetes does not reflect changes in iron stores.

In a randomized controlled study conducted by El Hajj et al. in Lebanon, 88 nonobese diabetic patients with vitamin D deficiency or insufficiency were randomly divided into two groups; the study group was given 30,000 IU of cholecalciferol per week for six weeks. In the Vit D-given group, CRP and TNF-α levels were found to be significantly lower than in the placebo group. However, the difference between IL-6 levels was not found to be statistically significant [26]. Although a clear mechanism is not well understood, there are several explanations for reducing circulating CRP with Vit D supplementation. It is known that NF-kB activation participates in the induction of endogenous CRP by increasing the effects of the signal transducer and activator of transcription-3 (*STAT3*) [27]. The biologically active form of Vit D can inhibit NF-κB activation by upregulating the NF-kB inhibitor (*IκB-α*). Vit D also inhibits STAT3 expression [28]. As a result, it has been concluded that Vit D supplementation can suppress CRP via NF-κB and *STAT3* signaling. In our study, the CRP and TNF-α levels were significantly higher in the Vit D deficiency and Vit D insufficiency groups compared to the normal Vit D group (Table 2).

In a cross-sectional study conducted by Zhang et al., 199 prediabetes patients were compared with 259 normal patients. The CRP levels were higher in the prediabetes group with Vit D deficiency and insufficiency, but no statistically significant differences were found between the IL-6 and TNF-α values [1]. In our study, the TNF-α and IL-6 levels in both the preDM and DM groups were found to be statistically significantly higher in patients with Vit D deficiency and insufficiency compared to normal ones (Table 3 and Table 4). The CRP levels were significantly higher in the preDM and DM groups compared to the nonDM group (Table 1).

The effects of Vit D may also be achieved by targeting MAPK signaling pathways, and the signaling of pro-inflammatory mediators may be caused by the activation of MAPK. The study by Ding et al. investigated the effects of Vit D on macrophage-mediated inflammatory responses in cultured human adipocytes, particularly its signaling pathways [11]. A macrophage-conditioned (MC) medium (with 25% adipocyte medium) markedly inhibited the NF-κB inhibitor kB-α (*IkB-α*) protein expression and increased the NF-κB levels. The addition of 1,25(OH)2D3 to the medium increased *IκB-α* expression and decreased NF-κB phosphorylation. Thus, macrophage-induced activation of NF-κB signaling was inhibited. 1,25(OH)2D3 can also blunt the MAPK signal by downregulating phosphorylated p38 MAPK and phosphorylated ERK1/2, which are the conventional MAPKs [11]. Several studies suggested a positive regulatory role of the ERK1/2 signaling module in glucose-stimulated insulin secretion in pancreatic β-cells [29]. In our study, serum MAPK levels were found to be statistically significantly higher in the Vit D-deficient and -insufficient groups compared to normal Vit D group in both the preDM and DM groups (Table 3 and Table 4). The fact that Vit D exerts its effects on pro-inflammatory markers IL1-β, IL-6 and TNF-α targeting NF-κB and MAPK signaling pathways may be the reason for higher levels of NF-κB and MAPK in the preDM and DM groups with deficient and insufficient Vit D levels in our study.

### Strengths and Limitations of the Study

Our study investigated the association between serum Vit D levels and the pro-inflammatory markers in new-onset T2DM and prediabetes patients without diabetic complications. In addition, we compared this association with the effects of Vit D on healthy subjects without any chronic disease. We also compared the pro-inflammatory markers between Vit D deficiency, Vit D insufficiency and normal Vit D in both prediabetes and T2DM groups. Aside from IL1-β, IL-6, IL-8 and TNF-α, we used NF-κB and MAPK for these comparisons. These factors make our study more valuable than previous studies that point to the same effects of serum Vit D levels. However, our study has its weaknesses as well as strengths. Firstly, all of the participants in this study were selected from a single province, and the population was underrepresented. Because this was a cross-sectional investigation, we were unable to conclude whether the logistic regression analysis revealed a relationship between diabetes and all inflammatory parameters. This study was based on a single blood measurement, which may not accurately represent the concentrations of circulating inflammatory parameters throughout time, and the serum IL-1β, IL-6, IL-8, TNF-α, NF-κB and MAPK levels should be assessed at different stages to better understand their function in the pathogenesis of T2DM. As a result, more large-scale, prospective studies with larger sample sizes must be performed to confirm the link between blood levels of pro-inflammatory parameters and serum Vit D levels in new-onset T2DM and prediabetes. The circulating serum Vit D level reflects exposure to Vit D from different sources such as the use of vitamin D supplements, diet or cutaneous biosynthesis. Studies investigating the relationship between serum Vit D levels and T2DM like our study can cause biases at this point. Although we excluded participants who used vitamin D preparations, calcium preparations or other vitamin preparations in the past three months, the intratrial serum 25(OH)D level measurement instead of serum Vit D level can be a way to prevent those biases. In the study conducted by Dawson-Hughes et al., adults with prediabetes who maintained higher intratrial serum 25(OH)D levels had a reduced risk of diabetes, with the greatest risk reduction occurring at intratrial 25(OH)D levels of 125 nmol/L and a partial risk reduction at levels of 100–124 nmol/L [30].

## 5. Conclusions

In conclusion, patients with Vit D insufficiency and deficiency were found to have higher levels of pro-inflammatory markers in new-onset T2DM and prediabetes. Since it is known that chronic inflammation can lead to the development of diabetic complications, Vit D-related inflammation is an important topic that needs to be investigated.

## Figures and Tables

**Table 1:** Distribution of Vit D levels, pro-inflammatory markers and other parameters between groups.

	Nondiabetes (NonDM) (*n* = 113)		Prediabetes (PreDM) (*n* = 84)		Diabetes (DM) (*n* = 94)		*p* Values		
	Mean	SD	Mean	SD	Mean	SD	NonDM vs. PreDM	NonDM vs. DM	PreDM vs. DM
Age (years)	36.54	10.48	44.75	9.76	50.08	9.34	**<0.001**	**<0.001**	**0.001**
BMI (kg/m^2^)	25.85	5.52	28.88	5.55	29.34	4.82	**<0.001**	**<0.001**	1.00
Waist circumference (cm)	85.97	13.33	95.86	12.64	102.99	16.08	**<0.001**	**<0.001**	**0.003**
25(OH)D3 level (ng/mL)	23.81	12.33	21.55	9.78	19.35	13.36	0.503	**0.001**	**0.014**
Calcium (mg/dL)	9.29	0.35	9.27	0.51	9.29	0.41	0.684	0.965	0.727
PTH (pg/mL)	36.91	15.28	35.64	16.39	47.66	38.07	0.534	0.111	**0.034**
IL-1β (pg/mL)	25.58	4.97	33.87	4.71	32.75	12.97	**<0.001**	**<0.001**	0.138
IL-6 (pg/mL)	20.48	2.86	24.79	4.10	24.20	9.62	**<0.001**	**<0.001**	0.096
IL-8 (pg/mL)	46.08	7.24	50.21	13.14	56.14	17.13	0.247	**<0.001**	0.250
TNF-α (pg/mL)	29.68	3.78	37.98	5.01	44.24	7.55	**<0.001**	**<0.001**	**<0.001**
NFκB (ng/mL)	0.48	0.08	0.54	0.11	0.70	0.22	**0.011**	**<0.001**	**<0.001**
MAPK (pg/mL)	160.53	22.20	194.60	27.70	230.88	30.99	**<0.001**	**<0.001**	**<0.001**
CRP (mg/L)	1.89	1.96	3.28	4.14	5.37	6.35	**0.007**	**<0.001**	**0.007**
Fibrinogen (mg/dL)	304.86	76.30	323.18	63.28	366.97	97.82	0.86	**<0.001**	**0.019**
Ferritin (ng/mL)	76.21	71.73	78.66	79.04	116.74	99.91	0.912	**0.003**	**0.005**

NonDM: nondiabetes mellitus, preDM: prediabetes, DM: diabetes mellitus, BMI: body mass index, 25(OH)D3: 25-hydroxyvitamin D3, PTH: parathyroid hormone, IL-1β: interleukin-1β, IL-6: interleukin-6, IL-8: interleukin-8, TNF-α: tumor necrosis factor-α, NF-κB: nuclear factor kappa-B, MAPK: mitogen-activated protein kinase, CRP: C-reactive protein. *p* < 0.05 values are shown in bold. ANOVA Bonferroni and Kruskal–Wallis Dunn tests were used.

**Table 2:** Distribution of pro-inflammatory markers and other biochemical parameters by Vit D levels.

	Vit D Deficiency (<20 ng/mL) (*n* = 142)		Vit D Insufficiency (20–30 ng/mL) (*n* = 88)		Normal Vit D (≥30 ng/mL) (*n* = 61)		*p* Values		
	Mean	SD	Mean	SD	Mean	SD	Normal Vit D vs. Vit D Ins.	Normal Vit D vs. Vit D Def.	Def vs. Vit D Ins.
FBG (mg/dL)	128.24	75.64	106.32	58.17	94.83	34.20	0.066	**<0.001**	0.195
Hemoglobin A1c (%)	6.94	2.27	7.35	9.88	6.18	1.53	0.268	**0.046**	0.383
Insulin (µIU/mL)	15.09	9.79	13.98	7.83	13.25	14.77	0.540	**0.001**	**0.012**
IL-1β (pg/mL)	34.94	8.49	27.86	6.58	22.57	7.47	**<0.001**	**<0.001**	**0.002**
IL-6 (pg/mL)	26.09	5.94	21.52	4.77	17.32	5.20	**<0.001**	**<0.001**	**<0.001**
IL-8 (pg/mL)	57.11	13.01	46.67	10.05	40.23	10.15	**<0.001**	**<0.001**	**0.002**
TNF-α (pg/mL)	40.10	7.90	35.66	6.05	30.50	8.27	**<0.001**	**<0.001**	**0.002**
NFκB (ng/mL)	0.61	0.19	0.56	0.14	0.47	0.15	0.132	**<0.001**	**0.002**
MAPK (pg/mL)	210.96	32.19	182.15	28.11	165.84	50.25	**<0.001**	**<0.001**	0.177
CRP (mg/L)	4.29	5.548	2.39	2.642	2.84	4.201	**0.005**	**0.007**	0.771
Fibrinogen (mg/dL)	344.66	91.78	316.26	73.15	315.93	77.79	**0.055**	0.105	0.959
Ferritin (ng/mL)	98.95	90.96	79.85	81.05	83.60	77.38	0.108	0.253	0.797

Vit D: vitamin D, Ins.: insufficiency, Def: deficiency, FBG: fasting blood glucose, IL-1β: interleukin-1β, IL-6: interleukin-6, IL-8: interleukin-8, TNF-α: tumor necrosis factor-α, NF-κB: nuclear factor kappa-B, MAPK: mitogen-activated protein kinase, CRP: C-reactive protein. *p* < 0.05 values are shown in bold. Kruskal–Wallis Dunn test was used.

**Table 3:** Distribution of inflammation markers and acute phase reactants by Vit D levels in the prediabetes group.

	Vit D Deficiency (<20 ng/mL) (*n* = 35)		Vit D Insufficiency (20–30 ng/mL) (*n* = 34)		Normal Vit D (≥30 ng/mL) (*n* = 15)		*p* Values		
	Mean	SD	Mean	SD	Mean	SD	Normal Vit D vs. Vit D Ins.	Normal Vit D vs. Vit D def.	Vit D Def vs. Vit D Ins.
IL-1β (pg/mL)	36.71	2.49	32.83	2.46	29.29	7.83	**<0.001**	**<0.001**	0.636
IL-6 (pg/mL)	27.88	2.31	23.24	2.09	20.83	5.69	**<0.001**	**<0.001**	0.503
IL-8 (pg/mL)	58.22	12.25	45.87	8.16	40.72	14.43	**<0.001**	**<0.001**	0.545
TNF-α (pg/mL)	40.80	2.40	37.20	1.63	32.83	9.23	**<0.001**	**<0.001**	0.396
NFκB (ng/mL)	0.56	0.09	0.55	0.08	0.48	0.19	0.970	0.085	0.115
MAPK (pg/mL)	212.05	15.76	185.35	11.76	173.42	48.11	**<0.001**	**<0.001**	0.849
CRP (mg/L)	3.66	3.44	2.86	3.37	3.36	6.90	0.432	0.825	0.706
Fibrinogen (mg/dL)	331.94	67.29	316.62	59.05	317.21	64.88	0.320	0.466	0.977
Ferritin (ng/mL)	96.92	87.22	52.74	60.10	96.76	86.26	**0.028**	0.988	0.14

Vit D: vitamin D, Ins.: insufficiency, Def: deficiency, IL-1β: interleukin-1β, IL-6: interleukin-6, IL-8: interleukin-8, TNF-α: tumor necrosis factor-α, NF-κB: nuclear factor kappa-B, MAPK: mitogen-activated protein kinase, CRP: C-reactive protein. *p* < 0.05 values are shown in bold. Kruskal–Wallis Dunn test was used.

**Table 4:** Distribution of inflammation markers and acute phase reactants by Vit D levels in the diabetes group.

	Vit D Deficiency (<20 ng/mL) (*n* = 57)		Vit D Insufficiency (20–30 ng/mL) (*n* = 22)		Normal Vit D (≥30 ng/mL) (*n* = 15)		*p* Values		
	Mean	SD	Mean	SD	Mean	SD	Normal Vit D vs. Ins.	Normal Vit D vs. def.	Def vs. Ins.
IL-1β (pg/mL)	38.74	10.946	25.99	9.712	19.02	9.218	**<0.001**	**<0.001**	0.540
IL-6 (pg/mL)	28.33	7.958	20.41	8.760	13.37	5.785	**0.014**	**<0.001**	**0.033**
IL-8 (pg/mL)	61.59	16.061	51.19	16.571	41.70	11.251	**0.013**	**<0.001**	0.168
TNF-α (pg/mL)	47.26	5.203	42.54	5.179	34.64	9.998	**0.002**	**<0.001**	**0.047**
NFκB (ng/mL)	0.76	0.215	0.67	0.197	0.52	0.212	0.139	**<0.001**	**0.011**
MAPK (pg/mL)	241.21	16.278	218.95	16.491	207.58	62.651	**<0.001**	**0.007**	0.602
CRP (mg/L)	6.79	7.446	2.60	1.816	3.93	4.217	0.01	0.095	0.786
Fibrinogen (mg/dL)	379.07	107.85	351.18	82.635	342.50	69.801	0.775	0.638	0.971
Ferritin (ng/mL)	117.41	101.36	130.42	109.02	93.64	80.777	0.616	0.432	0.291

Vit D: vitamin D, Ins.: insufficiency, Def: deficiency, IL-1β: interleukin-1β, IL-6: interleukin-6, IL-8: interleukin-8, TNF-α: tumor necrosis factor-α, NF-κB: nuclear factor kappa-B, MAPK: mitogen-activated protein kinase, CRP: C-reactive protein. *p* < 0.05 values are shown in bold. Kruskal–Wallis Dunn test was used.

**Table 5:** Intergroup correlation analysis of inflammation markers according to Vit D levels.

	Vitamin D Levels					
	NonDM (*n* = 113)		PreDM (*n* = 84)		DM (*n* = 94)	
	r	*p* Value	r	*p* Value	r	*p* Value
IL-1β (pg/mL)	−0.693 ***	**<0.001**	−0.589 ***	**<0.001**	−0.629 ***	**<0.001**
IL-6 (pg/mL)	−0.706 ***	**<0.001**	−0.714 ***	**<0.001**	−0.605 ***	**<0.001**
IL-8 (pg/mL)	−0.708 ***	**<0.001**	−0.669 ***	**<0.001**	−0.444 ***	**<0.001**
TNF-α (pg/mL)	−0.493 ***	**<0.001**	−0.620 ***	**<0.001**	−0.588 ***	**<0.001**
NFκB (ng/mL)	−0.198 *	**0.036**	−0.230 *	**0.036**	−0.379 ***	**<0.001**
MAPK (pg/mL)	−0.624 ***	**<0.001**	−0.551 ***	**<0.001**	−0.434 ***	**<0.001**
CRP (mg/L)	0.000	0.997	−0.100	0.368	−0.258 *	**0.012**
Fibrinogen (mg/dL)	−0.146	0.126	−0.102	0.357	−0.162	0.12
Ferritin (ng/mL)	0.064	0.510	−0.104	0.359	−0.057	0.596

* *p* < 0.05, *** *p* < 0.001; Pearson and Spearman correlation tests were used. NonDM: nondiabetes mellitus, preDM: prediabetes, DM: diabetes mellitus, IL-1β: interleukin-1β, IL-6: interleukin-6, IL-8: interleukin-8, TNF-α: tumor necrosis factor-α, NF-κB: nuclear factor kappa-B, MAPK: mitogen-activated protein kinase, CRP: C-reactive protein.

**Table 6:** The regression analysis for diabetes group.

			95% C.I.	
	Sig.	Exp(B)	Lower	Upper
Age (years)	0.005	1.144	1.041	1.258
25(OH)D3 level (ng/mL)	0.049	1.081	1.000	1.169
TNF-α (pg/mL)	0.02	1.326	1.046	1.680
MAPK (pg/mL)	0.016	1.057	1.010	1.105
Constant	<0.001	<0.001		

25(OH)D3: 25-hydroxyvitamin D, TNF-α: tumor necrosis factor-α, MAPK: mitogen-activated protein kinase.

## Data Availability

The datasets used and/or analyzed during the current study are available from the corresponding author upon reasonable request.
